# Editorial Changes

**Published:** 1848-11

**Authors:** 


*Editorial Changes.*—Several changes in the medical editorial corps of this country have lately occurred. Prof. C. A. Lee has resigned connection with the New York Journal of Med. and Collat. Sciences, and is succeeded by Dr. Purple, who has heretofore assisted Dr. L. in his editorial labors. Of the distinguished ability of our friend, the late editor, it would be supererogatory to speak. We shall not be accused of being actuated by the prepossessions of friendship when we say, that few of those who are prominent as contributors to medical literature, possess talents and acquirements so peculiarly and eminently fitted for editorial success. During the five years of his dynasty, the New York Journal, (the management of which he reluctantly consented to assume, chiefly from respect to the memory of its founder, the lamented Fory,) has arrived at a degree of success in which the views of publishers, friends, and the profession at large, are satisfied. But, like others who have been induced to assume a similar trust in addition to other severe duties, he found that "it brought with it more of care and responsibility than he anticipated;" and, hence, like others also, in obedience to the dictates of self-preservation, he now "hands over the work, and the hard earned results of his experience to his successor," While we regret that the periodical medical literature of our country is to loose his valuable services, it is a subject of congratulation that his time and talents may now be devoted to labors, which, if not more useful, will be more satisfactory to himself, and add to his reputation monuments more enduring than the most successful efforts of the journalist. With regard to the Journal in the hands of its present editor, in the language of Dr. L. we would "express the hope that it may long continue to flourish, and contribute in a good degree in advancing the interests of science and humanity."

Our New York City contemporary, the Annalist, has, also, recently passed into new editorial hands, owing to a change of proprietors. The farewell address of its late editor, Dr. Wm. C. Roberts, came upon us quite unexpectedly, and, if we may judge from our own sentiments, we are sure has been perused by the readers of the Journal with not less regret, than surprise. In making this remark, we shall not be understood as desiring to intimate that the Journal will not be ably conducted hereafter. It is no imputation upon the merits of a new acquaintance that we part with an old friend with great reluctance. Without being able to claim the honor of a personal acquaintance with Dr. R., we have seemed to form with him, through the medium of our exchange, and many editorial courtesies, a friendly relation, which, on our part, we are loath to have sundered. His editorial writings have a fervor and sincerity which secure a warmer approbation than that which flows from the intellect alone. While their good sense commends them to the judgment, their heartiness wins upon the hearts of his readers. Dr. R. is succeeded by Dr. N. S. Davis, late of Binghamton, N. Y.

Another editorial change is the union of the two medical Journals published at St. Louis, Mo., the editors of each Journal being retained, making an aggregate of four. It is highly creditable to the editors of each of the Journals now merged, that while occupying rival positions as journalists, nothing ever transpired to mar their harmony and mutual good will. The editors of the two journals were, and still are connected with rival medical schools. With regard to this, they say in their announcement, that they cannot see in this circumstance "the slightest reason why they may not harmonize in the cause of science and humanity, to the promotion and advancement of which alone the journal will be devoted." Such being the spirit of the combination, with the guaranty afforded by the past, excellent results may be safely predicted.

